# Selection of Shear Horizontal Wave Transducers for Robotic Nondestructive Inspection in Harsh Environments

**DOI:** 10.3390/s17010005

**Published:** 2016-12-22

**Authors:** Sungho Choi, Hwanjeong Cho, Cliff J. Lissenden

**Affiliations:** Department of Engineering Science and Mechanics, The Pennsylvania State University, University Park, PA 16802, USA; szc5748@psu.edu (S.C.); huc146@psu.edu (H.C.)

**Keywords:** electromagnetic acoustic transducers (EMATs), magnetostrictive transducers (MSTs), shear horizontal (SH) guided waves, robotic nondestructive inspection

## Abstract

Harsh environments and confined spaces require that nondestructive inspections be conducted with robotic systems. Ultrasonic guided waves are well suited for robotic systems because they can provide efficient volumetric coverage when inspecting for various types of damage, including cracks and corrosion. Shear horizontal guided waves are especially well suited for robotic inspection because they are sensitive to cracks oriented perpendicular or parallel to the wave propagation direction and can be generated with electromagnetic acoustic transducers (EMATs) and magnetostrictive transducers (MSTs). Both types of transducers are investigated for crack detection in a stainless steel plate. The MSTs require the robot to apply a compressive normal force that creates frictional force coupling. However, the coupling is observed to be very dependent upon surface roughness and surface debris. The EMATs are coupled through the Lorentz force and are thus noncontact, although they depend on the lift off between transducer and substrate. After comparing advantages and disadvantages of each transducer for robotic inspection the EMATs are selected for application to canisters that store used nuclear fuel.

## 1. Introduction

**Figure 1:**
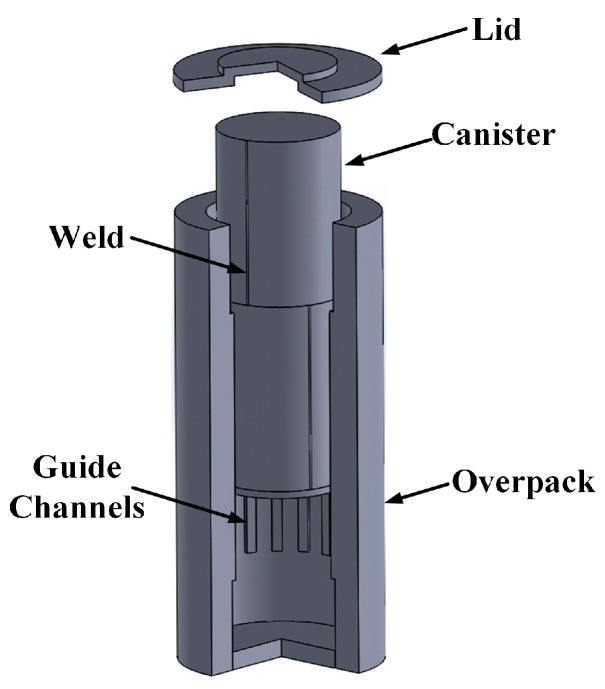
Cut-away view of vertical-axis dry storage cask.

Many nondestructive testing (NDT) and nondestructive inspection (NDI) applications in manufacturing, power generation, vehicle propulsion, and chemical processing involve harsh environments and/or geometric constraints that make robotically delivered NDT or NDI either preferred or required. The dry storage of used nuclear fuel is an excellent example [1,2,3,4]. Spent fuel rods are confined inside an austenitic stainless steel cylindrical canister (Figure 1) that is shielded and protected by a concrete and steel cask. The plenum between the cask and the canister enables convective cooling of the canister, but in many casks the air gap is very narrow, which effectively prevents direct access to a large portion of the canister’s external surface. While over time the canister cools and the radiation dose decreases, the combination of elevated temperature and radiation represents a harsh environment that could last for centuries. In this article, the dry storage of used nuclear fuel application is used as an example harsh environment with severe geometric constraints that guides the selection of transducers for robotically delivered NDI of a stainless steel canister. Based on numerical modeling of dry storage casks the highest temperature and largest gamma radiation dose in which the robotic inspection system could be expected to function are 177 °C and 27 krad/h, respectively. Note that neutron radiation is significantly lower than gamma radiation in this application. Transducer materials need to be capable of functioning at temperatures and radiation doses up to these values.

The cask type with the most constrained geometry has guide channels on the inside of the cask that maintain the general concentricity of the canister and the cask. The guide channels are nominally 150 mm wide and are separated by 214 mm gaps. The clearance between a guide channel and the canister is nominally 25 mm, but could be as small as zero. Thus, transducers and robots should operate in the 214 mm by roughly 50 mm wide gaps between guide channels. Moreover, transducers that make point measurements will not have access to a large portion of the canister external surface. Of course, there is no access to the canister internal surface.

The canister degradation mode of most significant concern is chloride induced stress corrosion cracking (CISCC) [1,2,3,4,5,6,7,8,9], which is most likely to occur in the heat affected zone of full penetration welds in the canister. Based on the thermal residual stress state [10,11,12], the cracks are expected to grow transverse to the weld line, or possibility along it. Many NDI techniques could be used to detect CISCC, e.g., magnetic particles, dye penetrant, eddy currents, bulk wave ultrasonics, but here ultrasonic guided waves are investigated in order to: (i) provide 100% volumetric coverage of the canister; (ii) make measurements with a robotic system; (iii) enable the use of noncontact transducers; and (iv) have the potential for both detection and sizing of cracks.

Shear horizontal (SH) waves are selected from the various types of guided waves [13] because they are sensitive to cracks oriented parallel to the wave vector as well as perpendicular to it [14]. In addition, SH waves are effectively actuated and sensed by electromagnetic acoustic transducers (EMATs) and magnetostrictive transducers (MSTs), which have strong potential for robotic applications. While the principle of operation of EMATs and MSTs will be reviewed in subsequent sections, Table 1 qualitatively assesses the transducer materials with respect to temperature and gamma radiation. Furthermore, the potential activation of elements such as cobalt should be considered for the selection of permanent magnets and magnetostrictive foil. Some quantitative information supporting Table 1 is that neodymium and samarium-cobalt alloys have Curie temperatures of 320 and 800 °C, respectively. The temperature-resistant neodymium alloys NUH and NEH have operating limits of 180 and 200 °C [15]. Likewise, polyimide and peek have temperature and gamma radiation resistances of 400 °C/5 × 10^9^ rad and 300 °C/1 × 10^9^ rad, respectively [16].

The subsequent Section 2, Section 3 and Section 4 describe the EMATs and MSTs to be investigated, and then mode/frequency selection for SH waves. Three different types of experiments to evaluate and compare the capabilities of the transducers are conducted. The functional assessment includes signal to noise ratio (SNR) as well as sensitivity to surface debris and surface roughness. Section 5 and Section 6 describe the laboratory experimental procedures and the experimental results, respectively, and are followed by the conclusions in Section 7.

## 2. Periodic Permanent Magnet (PPM) Electromagnetic Acoustic Transducers (EMATs)

**Figure 2:**
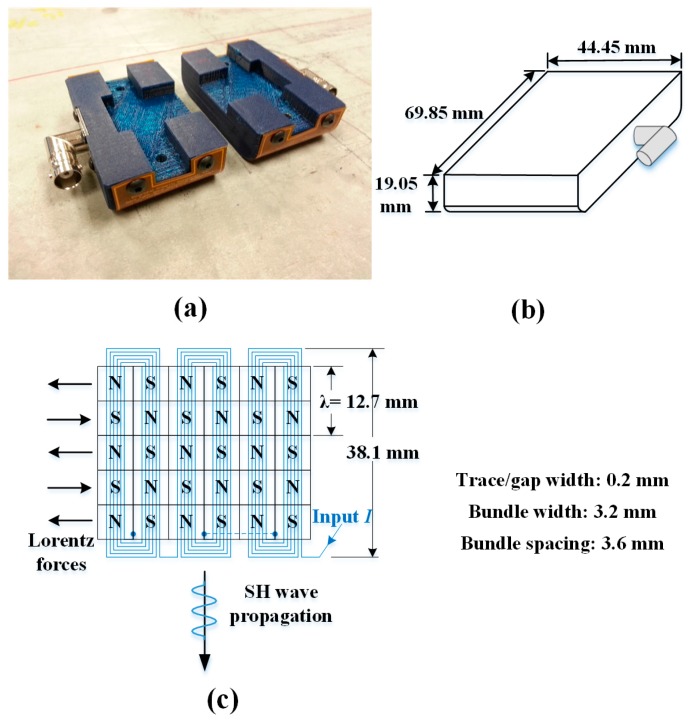
(**a**) Pair of PPM EMATs; (**b**) housing dimensions; and (**c**) layout of permanent magnets and electric coil.

Transduction in PPM EMATs is completely different from piezoelectric transduction, which requires a coupling medium between the transducer and the substrate. PPM EMATs can be effectively used to excite and receive a variety of types of ultrasonic waves, including SH waves in plates and torsional T (0,1) waves in pipes [17,18]. Moreover, the noncontact nature of EMATs is ideal for robotic delivery [19]. A pair of PPM EMATs, their dimensions, the layout of the permanent magnets and electric coil are shown in Figure 2. The transmitter and receiver are identical, each consisting of a periodic array of permanent magnets, a meander electric coil, and peripheral components. Importantly, they fit into the cargo bay of the robot currently being developed [4].

The transduction principal for PPM EMATs is based on the Lorentz force [17,18], which is the cross product of the eddy currents and static magnetic field near the surface of the waveguide. Thus, the waveguide material must be an electrical conductor. The periodicity of the array of magnets determines the wavelength of the generated SH mode, while the direction of the electric coil fixes the polarity of the SH wave propagation. Though the transduction efficiency of PPM EMATs is lower than for typical piezoelectric and magnetostrictive transducers [17], they offer noncontact transduction as well as suitable tolerance of radiation and high temperatures. Although the Lorentz force is larger in mild steel and aluminum, it is sufficiently large in stainless steel for NDT/NDI, even at high temperature [20].

## 3. Dry-Coupled Magnetostrictive Transducers (MSTs)

MSTs provide higher efficiency in exciting and receiving SH waves than PPM EMATs, but they require a coupling medium [21]. Each transducer comprises a magnet, a meandering electric coil, and a magnetostrictive foil such as remendur. Transduction with MSTs is based on the mechanical disturbance generated by strains associated with the motion of magnetic domains in the magnetostrictive foil, which is typically adhesively bonded to the sample surface. The wave in the foil is transmitted to the waveguide through the adhesive. However, in robotic inspection applications, the use of adhesively bonded foils is challenging at best. Therefore, dry frictional coupling between the foil and the substrate is investigated. A relatively large normal force is required to produce sufficient frictional forces to excite and receive SH waves. In dry storage casks the robot can apply normal force to the MST by a pneumatic piston or bladder restrained by the inside of the cask.

**Figure 3:**
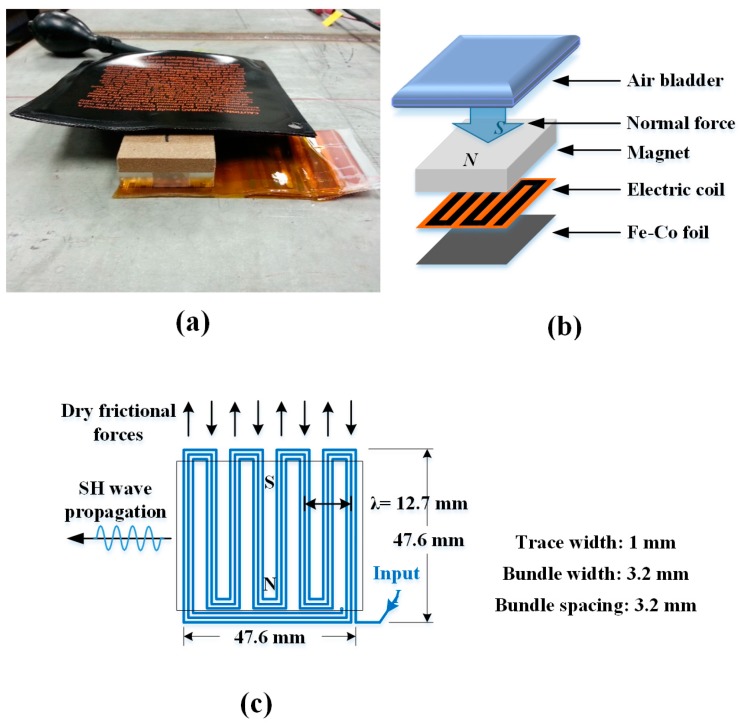
(**a**) MST prototype; (**b**) assembly of the MST; and (**c**) layout of permanent magnet and meandering electric coil.

The prototype MST, assembly of the MST, and layout of a permanent magnet and a meander electric coil are shown in Figure 3. The transmitter and receiver both have the same dimensions and configuration, and fit into the cargo bay in the robot. The spatial period of the meander coil determines the wavelength of SH wave mode. Figure 3a shows the MST with an air bladder on top, which must fit on the robot and transmit the normal force to the cask.

## 4. Mode/Frequency Selection of Shear Horizontal (SH) Waves

Since SH waves are multimodal and dispersive, mode/frequency selection is important for successful NDI. From a signal processing perspective it is desirable to excite a single mode, but often this is not possible given the finite size of the transducer. The selected mode should have good excitability/receivability with EMATs and MSTs as well as sensitivity to surface breaking cracks.

**Figure 4:**
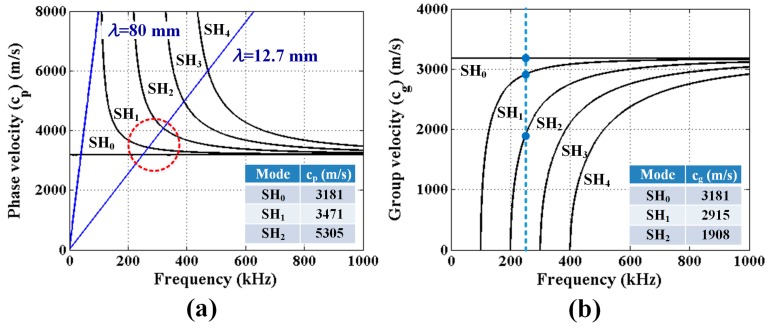
(**a**) Phase; and (**b**) group velocity dispersion curves for SH modes of a 15.9 thick stainless steel plate.

Propagating SH waves are given by the relationship between wave speed and frequency shown by dispersion curves [13]. The SH mode phase velocity and group velocity dispersion curves for a 15.9 mm thick stainless steel plate shown in Figure 4 are based on a mass density of 8000 kg/m^3^ and a measured shear wave speed of 3181 m/s. The fundamental mode, SH_0_, is often preferred because it is the one SH mode that is nondispersive. The blue lines in Figure 4a represent activation lines for EMATs and MSTs, and the slope of the activation line is the generated wavelength, which corresponds to the magnet spacing of the EMAT and the coil spacing of the MST. The intersection of the activation line and dispersion curves indicates the preferential excitation points for transducers having that wavelength. However, due to the finite size of the transducer and the finite frequency bandwidth of the toneburst pulse sent to the transducer there is a zone of excitation, rather than just a point. Thus, it is common for a transducer to excite secondary modes in addition to the preferred mode due to the source influence [13] as suggested by the red circle shown in Figure 4a around the SH_0_, SH_1_, and SH_2_ modes.

In order to ensure excitation of a single SH mode it would be necessary to excite the SH_0_ mode below the lowest cutoff frequency, which would require a wavelength of at least 80 mm. This would require a very large transducer and result in low defect sensitivity. Thus, we selected a wavelength of 12.7 mm with an excitation frequency of 250 kHz as a compromise between transducer size, crack sensitivity, and the presence of multiple modes. At this wavelength and frequency, SH_0_, SH_1_, and SH_2_ modes are expected to be generated as indicated inside the red dashed circle in Figure 4a. If the propagation distance is relatively short, the wave packets for the different modes will not have time to separate given their similar group velocities identified in Figure 4b.

Defect sensitivity of a given wave mode is dictated by its wave structure (i.e., displacement profile through the plate thickness). The particle displacement profiles for SH_0_ and SH_1_ modes are constant and vary linearly, respectively, making both modes sensitive to the surface breaking cracks of interest.

## 5. Experimental Procedures

Three different types of experiments to evaluate and compare the capabilities of the EMATs and MSTs are conducted:

- Test 1 to identify the effect of normal force applied to the transducers during SH wave measurements;
- Test 2 to examine how surface debris affects the performance of the transducers; and
- Test 3 to analyze the influence of surface roughness on the transduction efficiency of the transducers.

**Figure 5:**
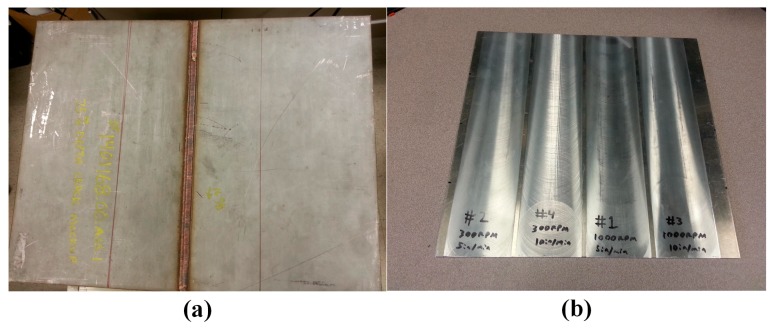
(**a**) A 12.7 mm thick 304 stainless steel plate; and (**b**) a 3 mm thick 2024 aluminum plate having four different degrees of surface roughness.

**Figure 6:**
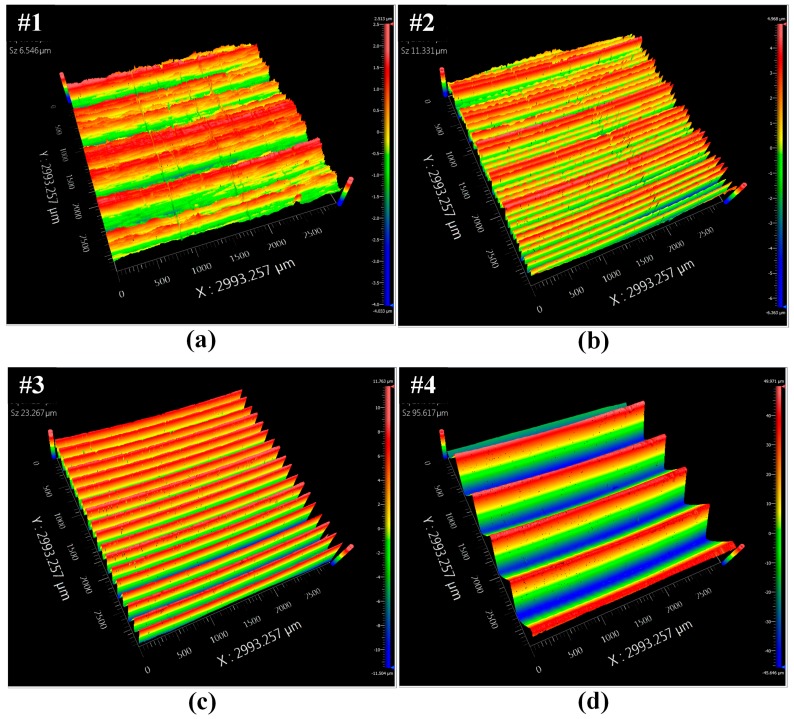
Typical profilometry images of each surface having different roughness conditions: (**a**) #1; (**b**) #2; (**c**) #3; and (**d**) #4.

The 12.7 mm thick 304 stainless steel plate shown in Figure 5a is used for Tests 1 and 2. While the plate has a full penetration weld, the weld is not part of these experiments. Dry storage canister thicknesses are 15.9 and 12.7 mm, and Figure 4 shows dispersion curves for a 15.9 mm thick plate. The predicted SH_1_ and SH_2_ phase and group velocities for a 250 kHz excitation frequency and both plate thicknesses are shown in Table 2 for comparison. Note that the cutoff frequency of the SH_2_ mode in a 12.7 mm thick plate is larger than 250 kHz. The 2024 aluminum plate shown in Figure 5b, having four different degrees of surface roughness was available for our use in Test 3. The surface treatments were made by mechanical milling at different spindle and feed speeds. In this 3 mm thick plate, the cut-off frequency for the SH_1_ mode is 520 kHz, which means that for a 250 kHz excitation only the SH_0_ mode will be generated. The surface roughness was measured using optical profilometry. Typical profilometry images of each surface are shown in Figure 6. The average surface roughness was determined from these images by averaging three micrographs from each surface, and is reported in Table 3.

**Figure 7:**
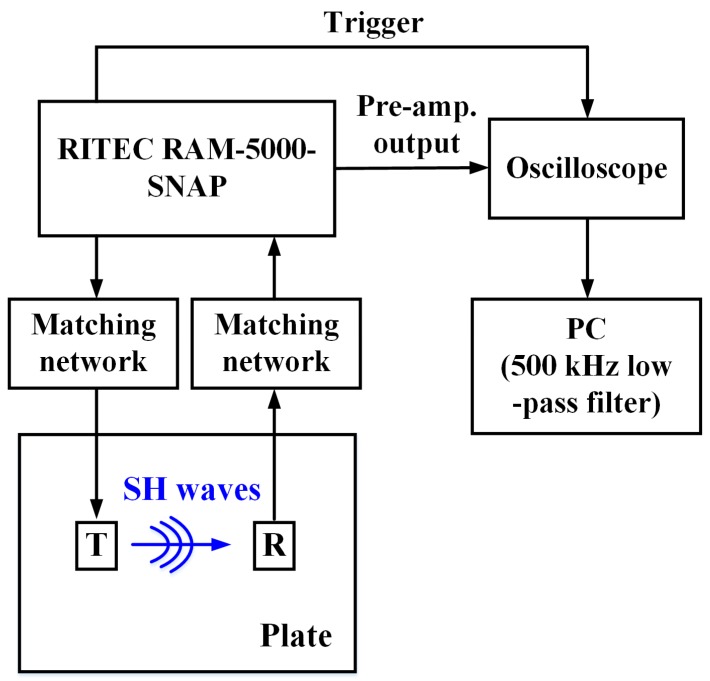
Schematic of the experimental setup for measuring SH waves using EMATs and MSTs.

A schematic of the experimental setup for measuring SH waves using EMATs and MSTs is shown in Figure 7. The RITEC RAM-5000-SNAP is used to provide a high-power electric tone-burst signal in the form of pure sine wave with a 1200 V peak-to-peak voltage and a 250 kHz central frequency. One cycle is sent to the MST and five cycles are sent to the EMAT in order to have similar as-received wave periods. The high-power signal passes through a matching network (TEM-128, RITEC, Warwick, RI, USA), and then to the transmitter to excite SH waves in the plate. After the receiver collects the SH waves, the acquired signal goes through a matching network (REMP-128, RITEC) and a pre-amplifier in the RITEC RAM-5000-SNAP. The pre-amplified signal is displayed on an oscilloscope (MSO 2014, Tektronix, Beaverton, OR, USA) and recorded. For post-processing, a 500 kHz digital low-pass filter is applied to the recorded signal to eliminate high frequency noise.

**Figure 8:**
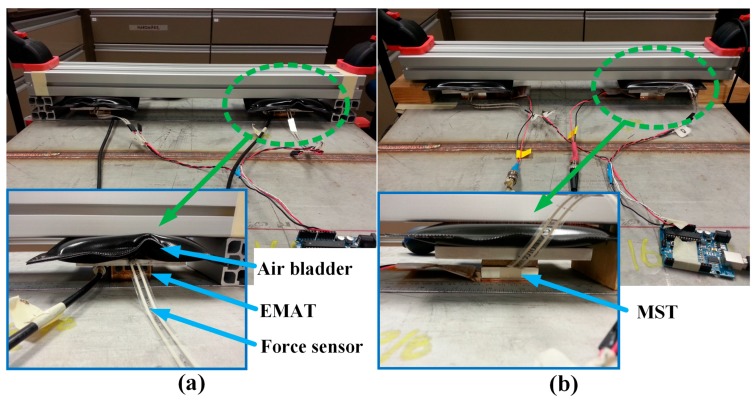
Photos of: (**a**) the EMAT; and (**b**) MST test setup on a stainless steel plate for analyzing the effect of normal force applied on the transducers on SH wave measurements.

Photos of the EMAT and MST test setup on the stainless steel plate for Test 1 are shown in Figure 8. In order to apply normal force to the transducers, an air bladder is placed in the 50 mm gap between the transducer and a restraint structure. Then, resistor type force sensors (FlexiForce A201, Tekscan, South Boston, MA, USA) are inserted underneath the air bladder to measure the applied normal force.

The experimental variable to be evaluated in each of the tests is summarized in Table 4. In Test 1, the normal force applied to the transducer varies from 0 to 267 N in 22.25 N increments with ±4.45 N accuracy. The wave propagation distance is 360 mm, which results in clear separation of the SH_0_ and SH_1_ mode packets in the A-scan. The subsequent tests are conducted at 0 and 222.5 N for EMATs and MSTs, respectively. These forces were determined after taking into account the effect of the force on SH wave measurements. In many applications there is potential for various types of debris to be present on the surface of the plate or shell. Therefore in Test 2 glass beads 45 to 850 μm in diameter, representative of surface debris, are placed between the receiver and the plate (but not at the transmitter). Only in Test 2, the received signals are averaged 16 times to check whether system noise could be reduced. Test 3 is conducted on the four different surface roughnesses shown in Figure 6. All measurements are repeated five times for each condition.

## 6. Experimental Results and Discussion

### 6.1. Test 1: Effect of Normal Force Applied to the Transducers during SH Wave Measurements

**Figure 9:**
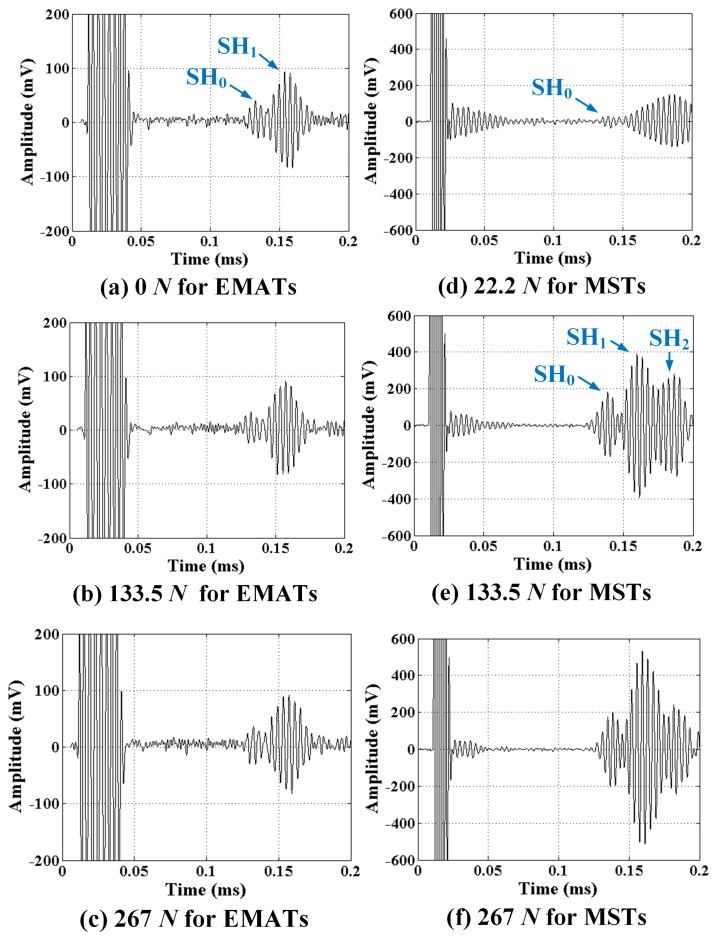
SH wave signals at different normal forces of: (**a**) 0; (**b**) 133.5; of (**c**) 267 N for EMATs; and (**d**) 22.2; (**e**) 133.5; and (**f**) 267 N for MSTs. Note that the signal prior to 0.04 ms is electromagnetic interference.

Typical SH wave signals received after propagating 360 mm are shown in Figure 9 for different normal forces applied to EMATs and MSTs. The strongest signals clearly show the presence of multiple modes (e.g., Figure 9e for MSTs with 133.5 N). The modes separate further as they propagate due to the group velocity differences. The higher order modes are more dispersive and hence have slower group velocities. Both EMAT and MST generate SH_0_ and SH_1_ modes, while the MST also generates the SH_2_ mode, which suggests that the EMAT has a more narrow excitation spectrum than the MST. The generation of fewer modes by the EMAT is preferred from a signal processing standpoint. The excitability/receivability of the SH_1_ mode is seen to be larger than the SH_0_ mode based on their amplitudes. This is expected based on their wavestructures and the transducer being located on the surface. In terms of SNR, the MSTs are superior to EMATs: 13.4 (SH_0_) and 35.3 (SH_1_) for MSTs, and 2.2 (SH_0_) and 5.1 (SH_1_) for EMATs at 267 N normal force. It is obvious in Figure 9 that the normal force affects the MST but not the EMAT, which is just as obvious from the transducer principle of operation. The MST relies on dry coupling based on the friction force created by the normal force. On the other hand, the EMAT operates on the Lorentz force, which is independent of the normal force. It is worth noting that when the MST is weakly coupled, i.e., by the 22.2 N force, the wave modes SH_1_ and SH_2_ are indistinguishable, as shown in Figure 9d.

**Figure 10:**
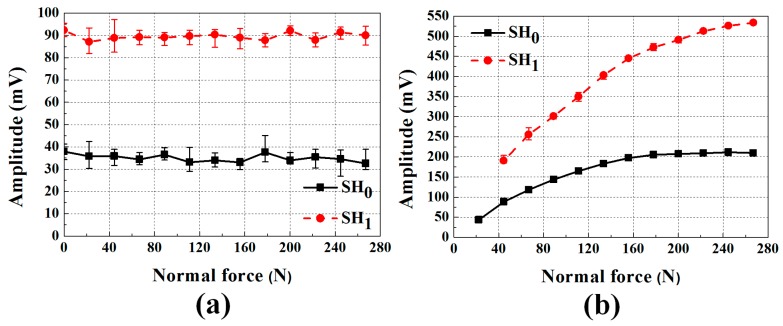
The averaged peak amplitudes of each mode for: (**a**) EMATs; and (**b**) MSTs as a function of the applied normal force. The EMATs are independent on the normal force, while the MSTs are not.

The affect of normal force on the transducers is more clearly shown in Figure 10. The peak amplitudes of the SH_0_ and SH_1_ modes for EMATs and MSTs are shown in Figure 10 as a function of the applied normal force, where each symbol is the average of five measurements and error bars represent the data range. As expected, the MSTs are highly dependent on the normal force, however the normal force dependence is much different for each mode. SH_0_ increases slowly with normal force to an asymptote, while SH_1_ increases more rapidly. Thus, if the MSTs are used to determine wave mode amplitudes it is important to accurately know the normal force value. Figure 10 suggests that each mode has a different normal force required to develop full frictional coupling, e.g., the SH_0_ mode becomes fully coupled at approximately 160 N, while the SH_1_ mode does not become fully coupled until at least 280 N. Therefore, normal forces from 40 to 280 N result in a broader range of SH_1_ amplitudes than for the SH_0_ mode.

### 6.2. Test 2: Influence of Surface Debris on SH Wave Measurements

**Figure 11:**
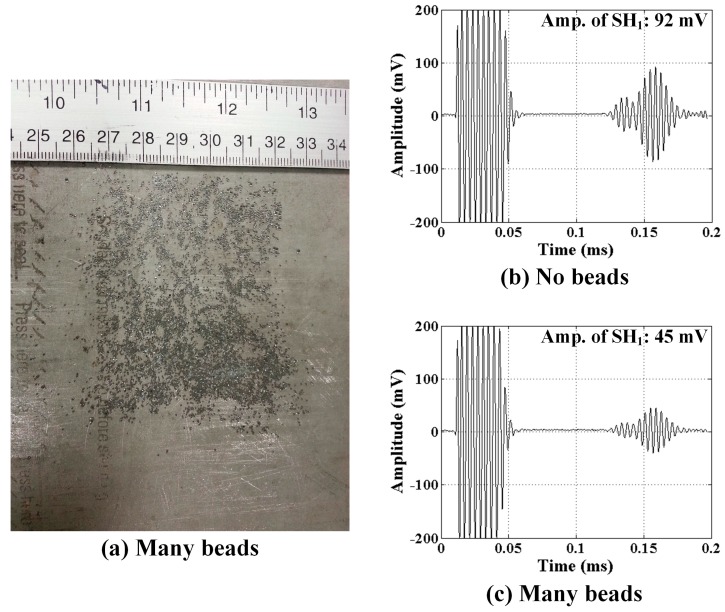
(**a**) Distribution of many glass beads between the EMAT receiver and the substrate; and SH wave signals received for: (**b**) no beads; and (**c**) many beads.

**Figure 12:**
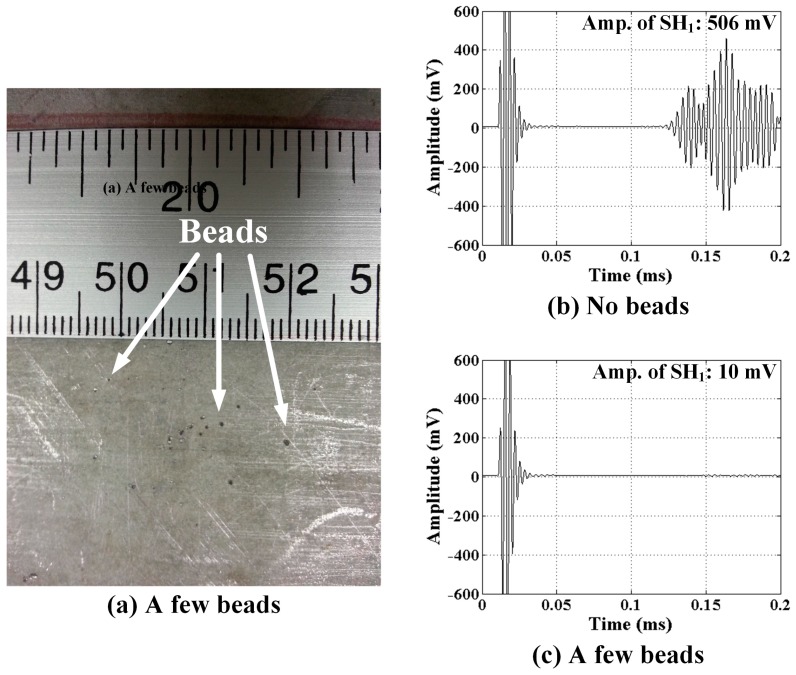
(**a**) Distribution of a few glass beads between the MST receiver and the substrate; and SH wave signals received for: (**b**) no beads; and (**c**) a few beads.

The distributions of glass beads placed on the sample surface for EMAT and MST tests are quite different to emphasize the effect that surface debris has on the transducer signals. In qualitative terms, many glass beads were used for EMAT tests while just a few were used for MST tests as shown in Figure 11a and Figure 12a, respectively. The effect of surface debris on the EMAT is to create “lift off” between the transducer and the surface. As the lift off increases the eddy currents in the substrate decrease, therefore decreasing the received signal. On the other hand, for the MST the surface debris reduces the frictional force that couples the transducer to the substrate.

The affect of the glass beads on received signals from the EMAT and MST are shown in Figure 11 and Figure 12, respectively, where all signals shown are a result of 16 averages. Analogous results are expected for glass beads at the transmitter. Received signals for both a bare substrate (no beads) and a substrate with beads are shown. It is clear by comparing Figure 11 and Figure 12 with Figure 9 that just 16 averages improve the SNR noticeably. There is no change in the waveform received by the EMAT, but the signal amplitude is decreased by approximately 50%. In this case the lift off is dictated by the diameter of the largest glass beads (850 μm), which is quite big. Debris having smaller size is expected to have a proportionally smaller effect. In contrast, the signal received by the MST is effectively nil when glass beads are present, even though the number of beads is very small (~20). Clearly, hard surface debris particles have a deleterious effect on MST operation due to the loss of frictional force coupling. Furthermore, the size of the debris particles is expected to play a relatively small role, allowing even smaller debris particles to prevent the MST from receiving signals. Thus, to deploy MSTs robotically it appears necessary for the robot to confirm that the surface is free of friction-reducing debris.

### 6.3. Test 3: Influence of Surface Roughness on Transduction Efficiency

**Figure 13:**
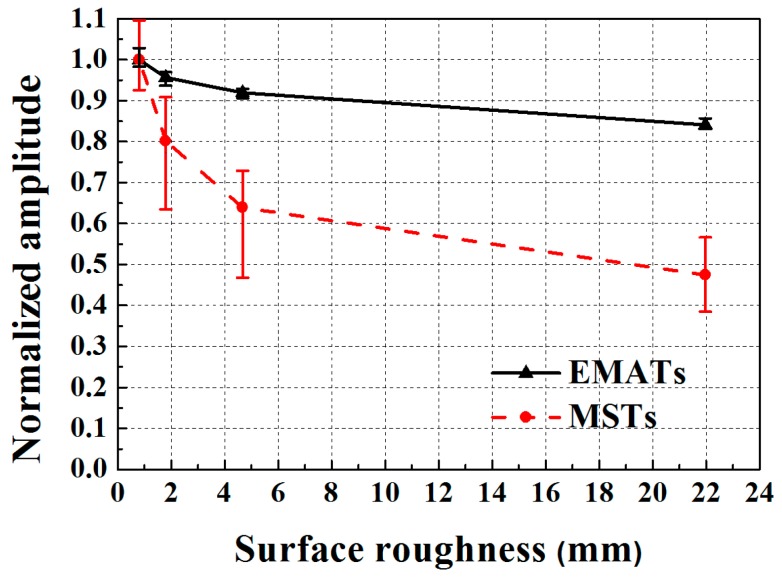
The normalized amplitudes of SH waves for EMATs and MSTs as a function of surface roughness.

Knowing that dry coupled MSTs depend on frictional forces to generate SH waves and that EMAT signals depend on lift off, the effect of surface roughness is investigated using a well defined roughness as described in Section 5. The decrease in normalized SH wave amplitude for EMATs and MSTs as a function of surface roughness is shown in Figure 13, where the amplitudes are normalized with respect to the amplitude for an average surface roughness of 0.811 μm. Both the average value and the data range of five measurements are shown in Figure 13. The MST exhibits significantly more amplitude variability and reduction than the EMAT. Clearly, the most significant amplitude reduction occurs for surface roughness below 5 μm. For a 22 μm surface roughness, the SH wave amplitudes are reduced to 0.84 and 0.47 for EMATs and MSTs, respectively. Figure 6 indicates that the contact area between the MST and the substrate decreases as surface roughness increases, which means that there is less area over which the frictional coupling can occur. Obviously, the MST is much more sensitive to surface roughness than the EMAT, which is a consideration when wave amplitude features are to be used for NDI/NDT.

### 6.4. Summary

The experimental observations are summarized in Table 5 in terms of the advantages and disadvantages of each transducer. A remarkable advantage of EMATs is that they are significantly less sensitive to surface debris and surface roughness than MSTs. Based on the EMAT principle of operation, only the increment of lift off induced by the surface conditions causes the amplitude reduction of the SH wave signal. However, the lower EMAT amplitude and SNR are noteworthy disadvantages. SNR can be improved by averaging. Likewise, placing a thin film on the EMAT surface and applying a small normal force can maintain consistent liftoff.

In contrast to EMATs, MSTs have many opposite tendencies. They provide much higher amplitude and SNR than EMATs for SH waves, which is their main advantage. Meanwhile, in order to excite and receive SH waves through dry coupling, a relatively large normal force to produce sufficient frictional forces is required. In addition, the SH wave amplitude is highly dependent on the normal force value and the degree of surface roughness, while EMATs are not. Thus, it could be quite challenging to relate the wave amplitude to degradation in practical applications. MST operation is also severely limited on surfaces where friction-reducing debris are present.

## 7. Conclusions

Nondestructive robotic inspection of cracks can be accomplished using shear horizontal guided waves, which can be generated from periodic permanent magnet electromagnetic acoustic transducers (EMATs) or dry coupled magnetostrictive transducers (MSTs). Both types of transducers are appropriate for harsh environments that include high temperature and radiation. While the MSTs can provide a higher amplitude wave form, because they rely on coupling through a frictional force between the transducer and the substrate, they require a significant normal compressive force and are sensitive to both surface debris and surface roughness. Thus, surface conditions must be carefully considered for robotic inspections. However, EMATs are noncontact transducers and provide consistent transduction sensitive to the surface conditions only through the lift off, and there is no need to apply a normal force to the transducer (except possibly to maintain a uniform lift off). For these reasons, EMATs have been selected for the inspection of stainless steel canisters that store used nuclear fuel.

## Figures and Tables

**Table 1:** Material selection considerations for transducer components.

Transducer Component	Transducer Type	Purpose	Candidate Materials	Elevated Temperature	Gamma Radiation Resistance
Permanent magnets	EMAT and MST	Generate a static magnetic field	Rare earth alloys like neodymium, samarium cobalt	Select carefully	Good
Electrical coil	EMAT and MST	Provide an alternating electric field	Metal conductors like copper, silver	Good	Good
Backing	EMAT and MST	Electrical insulation for coil	Polymers like Polyimide, peek	Select carefully	Select carefully
Magneto-strictive foil	MST	Energy conversion	Ferrous metals and alloys like remendur, iron	Good	Good

**Table 2:** Comparison of wave speeds for 250 kHz excitation frequency.

Mode	Plate Thickness (mm)	Phase Velocity (m/s)	Group Velocity (m/s)
SH_1_	15.9	3471	2915
	12.7	3675	2753
SH_2_	15.9	5305	1908
	12.7	-	-

**Table 3:** Surface roughness measured using optical profilometry.

	Surface Roughness (μm)		
Surface No.	Min.	Max.	Avg.
#1	0.776	0.869	0.811
#2	1.722	1.841	1.798
#3	4.64	4.707	4.675
#4	20.708	22.664	21.981

**Table 4:** Summary of experimental variables for each test.

	Variable	Values
Test 1	Normal force	0~267 N in 22.25 N increments
Test 2	Surface debris	zero and many glass beads (for EMATs) zero and a few glass beads (for MSTs)
Test 3	Surface roughness	0.811, 1.798, 4.675, and 21.981 μm

**Table 5:** Summary of the experimental observations.

	EMATs	MSTs
Advantages	- Low sensitivity to surface debris and surface roughness	- High amplitude and SNR of SH wave signal
Disadvantages	- Low amplitude and SNR of SH wave signal - Requirement to maintain a uniform lift off to provide consistent transduction	- Requirement of a relatively large normal force to excite and receive SH waves through dry fractional coupling - SH wave amplitude dependence on the normal force value and the degree of surface roughness - Limited operation on the surface where friction-reducing debris are present
